# RIZ1: a potential tumor suppressor in glioma

**DOI:** 10.1186/s12885-015-2023-1

**Published:** 2015-12-21

**Authors:** Chenran Zhang, Qiubei Zhu, Hua He, Lei Jiang, Qiang Qiang, Liuhua Hu, Guohan Hu, Ying Jiang, Xuehua Ding, Yicheng Lu

**Affiliations:** 1 Department of Neurosurgery, Changzheng Hospital, Second Military Medical University, Shanghai, 200003 China; 2 Department of Otolaryngology, Changzheng Hospital, Second Military Medical University, Shanghai, 200003 China; 3 Department of Neurology, Huadong Hospital, Fudan University, Shanghai, 200040 China; 4 Department of Cardiology, Ren Ji Hospital, School of Medicine, Shanghai Jiao Tong University, Shanghai, 200127 China

**Keywords:** RIZ1, Glioma, Prognosis, Tumor suppressor, Overall survival

## Abstract

**Background:**

Retinoblastoma protein-interacting zinc-finger gene 1 (RIZ1) displays strong tumor suppressive activities, and its expression is often silenced in many types of human tumors. However, the relationship between RIZ1 expression and glioma prognosis remains unclear.

**Methods:**

The dysregulation of RIZ1 was evaluated using real-time polymerase chain reaction, western blot, and immunohistochemical analysis of gliomas from 51 patients. Correlation analysis was performed to examine relationships between RIZ1 immunoreactivity, clinicopathological features, and patient prognosis. Also, human malignant glioma U87 and U251 cell lines were stably transduced with ectogenic RIZ1 using a lentiviral vector to investigate the effects of induced expression of RIZ1 on cell proliferation, cell cycle, and apoptosis.

**Results:**

Real-time polymerase chain reaction and western blot analysis showed that RIZ1 was downregulated in high-grade gliomas compared with low-grade gliomas and normal brain tissue. Immunohistochemistry showed less RIZ1 labeling in high-grade gliomas than in low-grade gliomas. There was a negative correlation between RIZ1 and Ki-67 immunoreactivity. Clinicopathological evaluation revealed that RIZ1 expression was negatively correlated with tumor grade and patient age. Kaplan-Meier survival analysis showed a positive correlation between RIZ1 immunoreactivity level and progression-free and overall survival times. Multivariate analysis showed that high RIZ1 expression was an independent prognostic factor for patients with gliomas. Induced expression of RIZ1 in U87 and U251 cells reduced cell proliferation and increased apoptosis, and cell cycle analysis revealed that a majority of cells were arrested at G2-M. Moreover, transfection with a RIZ1 expression vector increased p53 and caspase-3 expression and decreased p-IKBα and p-AKT protein levels, suggesting that RIZ1 may stimulate p53-mediated apoptosis and inhibit p-IKBα and p-AKT signaling pathways.

**Conclusions:**

Our results suggest that high RIZ1 labeling is indicative of lower grades of gliomas and is associated with better progression-free and overall survival rates. Therefore, RIZ1 may be a promising therapeutic target for patients with gliomas.

## Background

Both anaplastic astrocytomas (World Health Organization (WHO) grade III) and glioblastoma multiformes (GBMs; WHO grade IV) are classified as high-grade gliomas (HGGs), whereas Low-grade gliomas (LGGs) constitute grades I and II tumors of astrocytic and grade II tumors of oligodendroglial lineage. Compared with LGGs, HGGs have the highest annual incident rate (0.005 %) and worst prognosis. Despite advanced therapies for gliomas, including surgery, radiation, and chemotherapy, the overall 5-year survival rate of GBM remains less than 5 % [1]. Thus, the discovery of new therapies for HGGs is urgently needed.

Previous studies of HGG pathology have focused on identifying abnormalities in genetic events and signaling pathways, characterising tumor stem cells and tumor immunology, and exploring novel therapeutic approaches. However, despite promising new therapeutics (e.g., gene therapy), an ideal therapeutic target for HGG remains undefined. Therefore, investigations of the molecular underpinnings of HGG could provide insights into the malignancy of glioma cells, the mechanisms underlying glioma development, and the prediction of glioma prognosis.

The retinoblastoma protein-interacting zinc-finger gene 1 (RIZ1), which is closely related to cancer genesis, is located within tumor suppressor gene clusters on chromosome 1q36. The RIZ1 gene encodes a methyltransferase protein containing a PR/SET domain [2–4]. Similar to p53, RIZ1 serves as a cancer suppressor, as its deletion is observed in various types of human cancers including breast cancer, liver cancer, colorectal cancer, neuroblastoma, chronic myelogenous leukemia, and malignant meningioma [5–9]. However, it remains unclear whether RIZ1 influences the progression of gliomas.

The aim of the current study was to assess the pattern of RIZ1 expression in gliomas and its correlation with clinicopathologic characteristics and patient outcomes. We found that RIZ1 expression is negatively correlated with glioma differentiation and can serve as a predictor of glioma prognosis.

## Methods

### Cell culture

Human HGG cell lines U87 and U251 were obtained from the Chinese Academy of Sciences (Shanghai, China). Both cell lines were grown in Dulbecco’s Modified Eagle’s Medium with 8 % fetal bovine serum (FBS), 100 U/ml penicillin G, and 100 μg/ml streptomycin. The human breast cancer cell line MCF-7 was purchased from American Tissue Culture Collection (Manassas, VA) and was cultured in the same medium with 10 % FBS, 100 U/ml penicillin, and 100 mg/ml streptomycin. All cells were cultured at 37 °C in humidified air with 5 % CO_2_.

### Patients and tissue samples

The study protocol was examined and approved by the Shanghai Changzheng Hospital Ethics Committees. Prior to surgery, all patients were given informed written consent based on the Declaration of Helsinki. There were 32 males and 19 females (male-to-female ratio = 1.68:1.0) enrolled in the study, and the median age was 46 years (range, 22–75 years). The follow-up period ranged from 3–64 months. Clinicopathologic characteristics of patients are summarised in Table 1. The median progression-free survival (PFS) and overall survival (OS) times were 7.5 months (1.0–48.6 months) and 18.3 months, respectively.

**Table 1:** Correlation between RIZ1 immunoreactivity and clinicopathologic features

		RIZ1 immunoreactivity (Median = 8.0)		
Features	N(*N* = 51, %)	<Median	≥Median	*P*-values
Gender				0.097
Male	32(62.7)	19	13	
Female	19(37.3)	6	13	
Age				0.027*
< 50 years	29(56.9)	12	17	
≥ 50 years	22(43.1)	13	9	
Tumor diameter				0.324
< 6 cm	32(62.7)	14	18	
≥ 6 cm	19(37.3)	11	8	
Extent of resection				0.436
Gross-total	39(76.5)	20	19	
Sub-total	12(23.5)	5	7	
Pathological grade				0.000*
Low grade	12(23.5)	1	11	
High grade	39(76.5)	24	15	
Ki-67 immunoreactivity				
< Median	24(47.1)			
≥ Median	27(52.9)			

* P < 0.05 was considered statistically significant

Table 1: Clinicopathological evaluation suggested that RIZ1 expression was associated with tumor grade (*p* = 0.000) and patient age (*P* = 0.027)

All 51 glioma samples (LGG (WHO I and II grade) 12cases, HGG (WHO III and IV grade) 39cases) were surgically obtained in the Department of Neurosurgery at Changzheng Hospital, Shanghai, China, from March 2008 to December 2012. Immediately after resection, samples were divided into two parts: one part was fixed in formalin, and the other part was snap-frozen at -80 °C. The formalin-fixed gliomas were histopathologically graded according to the 2007 WHO classification of tumors of the central nervous system.

Normal brain tissue (NBT) samples were obtained from two individuals who died due to traffic accidents and presented no prior pathological conditions. All specimens were re-examined and stored by the Changzheng Hospital Institutional Review Board. Tumor size, extent of surgical resection, and time free from recurrence were recorded.

### Protein extraction and western blotting

The western blot analysis was performed as described previously [10]. Briefly, proteins were extracted and transferred onto nitrocellulose membrane (Millipore, USA). Then, these protein-embedded membranes were blocked in 5 % non-fat milk in 0.1 M phosphate-buffered saline (PBS) for 1 h at room temperature. After wash out in PBS, these membranes were blotted with primary antibodies overnight and followed by incubation with a peroxidase-conjugated secondary antibody for 1 h in room temperature. Enhanced chemiluminescence (Bio-Rad, Hercules, CA, USA) were used to visualize blots. The primary antibodies used in this study included the rabbit polyclonal antibody to RIZ1 (Abcam, Cambridge, USA; at 1:1000 dilution), mouse monoclonal antibody to P53 (Santa Cruz, 1:1000), goat polyclonal antibody to caspase-3 (Santa Cruz, 1:1000) and β-actin (Santa Cruz Biotechnology, Santa Cruz, CA, USA; at 1:1000 dilution). pAkt (S473; cat # 05-736) antibody was procured from Upstate Cell Signaling (Lake Placid, NY). Antibodies against p-PI3K (Tyr458; cat 4228 s) were from Cell Signaling Technologies (Danvers, MA). The relative amount of protein was quantified by densitometry using Image J software.

### Real-time PCR analysis

RNA was extracted from frozen tissue specimens and glioma cell lines using the RNeasy mini kit (Qiagen, Valencia, CA, USA). Reverse transcription was conducted instantly after RNA extraction in the PrimeScript™ RT Master Mix (TAKARA, RR036A, Dalian, China). The qPCR was performed by using the SYBR® Premix Ex Taq™ (TAKARA, DRR041A, Dalian, China) in the 7500 fast qPCR machine (Applied Biosystems, Foster City, CA, USA). The qPCR protocol included four steps: 5 min at 95 °C, 40 cycles of 95 °C for 30 s, 55 °C for 30 s and 72 °C for 30 s. The forward and reverse primers for the RIZ1 amplification were 5′- AACATGTGCTGCGAGGACTT -3′ and 5′- TTCTTCCCTTTCCGGCTCTT -3′, respectively. The forward and reversed primer sequences for the β-actin were 5′- CCAAGGCCAACCGCGAGAAGAT -3′ and 5′- TTGCTCGAAGTC CAGGGCGA -3′, respectively.

### Immunohistochemistry staining

Formalin-fixed, paraffin-embedded samples were first sectioned into 3-mm slides. Then slides were deparaffined in xylol and dehydrated in concentration-graded ethanol series. After the inactivation of endogenous peroxidase activity, the sections were heated in microwave for 20 min in 1 mM EDTA buffer (pH 8.0) to retrieve antigen. The sections were then blocked with 1 % bovine serum albumin for 40 min. Then, primary RIZ1 antibody (ab3790, Abcam) at 1:50 or Ki-67 primary antibody (ab15580, Abcam) at 1:75 overnight were incubated with slides at 48C overnight. Next day, primary antibodies were washed out and slides were incubated with biotin-conjugated secondary antibody for 20 min at room temperature. The peroxidase-conjugated biotin–streptavidin complex (Dako) was used for visualization.

### Evaluation of immunostaining

All sections were examined microscopically and scored by two independent pathologists who were blinded to the clinical diagnosis. Both Ki-67 and RIZ1 labeling index were recorded. The labeling index was defined as mean percentage by counting the positive staining of tumor cells for 1000 cells in three most-labeled areas. For calculating labeling index, tissue slides were divided into five levels: (1) immunoreactivity completely absent (negative, 0 %); (2) <5 %; (3) <25 %; (4) <50 %; and (5) up to 100 %.

### Cell culture and transfection studies

The human glioblastoma cell lines U87 and U251 were cultured as previously described. RIZ1 expression vector pCMV-RIZ1 and its control pCMV-GFP were prepared as previously described [9]. The establishment of stable transfectant glioblastoma cell lines U87 and U251 with the pCMV-RIZ1 or pCMV-GFP respectively were conducted as described by the manufacturer (Lipofectamine 2000, Invitrogen). After 48 h of incubation, the stable transfectant was screened and established by flow cytometry. And the expression product was tested for RIZ1 expression by Western blot.

### Cell cycle assay

PCMV-RIZ1 or pCMV-GFP cells were harvested, centrifuged at 1000 r.p.m. for 5 min, resuspended in phosphate buffered saline and incubated with 0.2 mg/ml propidium iodide (PI) containing 1 mg/ml RNase A (Sigma) for 10 min in the dark. Distribution of the cell cycle was quantitated by flow cytometry analysis. Data were collected from three different experiments and analyzed by BD Cell quest computer program.

### Apoptosis assay

Cell pellets collected and centrifuged at 1000 rpm for 5 min were washed with PBS and resuspended in 200 μl 1X binding buffer, mixed with 5 μL of annexin V-FITC and 10 μL of PI according to manufacturer’s instructions, and then incubated at room temperature for 5 min in the dark. Subsequently, cells stained by Annexin V-fluorescein isothiocyanate (FITC)/ propidium iodide (PI) were analyzed using luorescence-activated cell sorter (FACS) analysis.

### BrdU

BrdU (5-bromo-2′-deoxyuridine) Cell Proliferation Assay (chemiluminescence) was performed as manufacture’s procedure (Roche, Germany). Briefly, cells (1 × 10^5^) under different treatments were plated onto 96-well plates. Then, incubate the various types of cell line with Brdu labeling solution for 24 h. After removal of the labeling solution and fixing the cells, incubate cells with anti-BrdU Detection Antibody. Washing and adding substrate solution to each well, measure the light emission of samples in a microplant luminometer. Three independent experiments were performed.

### Statistics

All statistical analysis was performed in the SPSS 21.0 (IBM, Endicott, NY USA). The graphs were generated by Graphpad Prism 5 (GraphPad Software, Inc., La Jolla, CA, USA). The Kruskal–Wallis test was used to compare the immunolabeling results between the glioma tissue grades. The correlation between RIZ1 and Ki-67 intensity was examined by Spearman’s correlation coefficient. Univariate survival analysis was performed using the Kaplan–Meier method and analyzed by the log-rank test to assess survival differences between groups. Six possible prognostic factors were analyzed by univariate analysis for potential association with survival. Factors with a result of *P* < 0.2 in the univariate analysis were included in multivariate analysis for potential association with survival. *P* value of <0.05 was considered statistically significant.

## Results

### Downregulation of RIZ1 in HGG and GBM cell lines

**Fig. 1:**
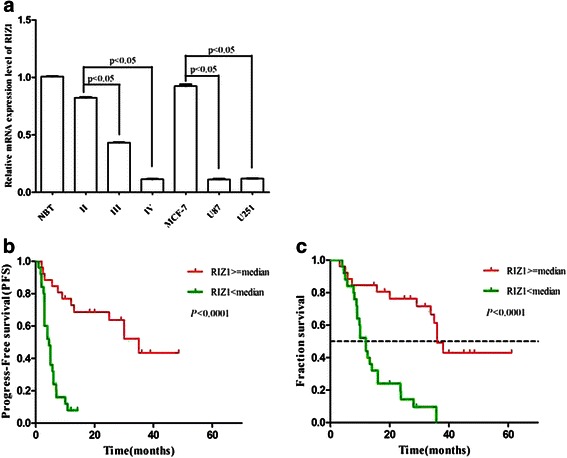
RT-PCR analysis of RIZ1 mRNA expression in different grade of gliomas as well as in U87 and U251 GBM cell lines (**a**). II: LGG, III and IV: HGG. Human breast cancer MCF-7 cells served as a positive control. PFS (**b**) and OS (**c**) associated with RIZ1 immunoreactivity in patients with gliomas. Three independent experiments were performed. *P < 0.05 as compared with control groups

**Fig. 2:**
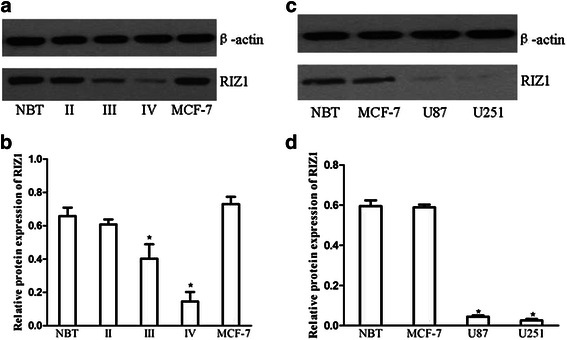
Western blot analysis of RIZ1 protein expression levels in NBT, LGGs (II), and HGGs (III and IV) as well as GBM cell lines (**a**, **c**). **b**, **d** Quantification of (**a**, **b**). Three independent experiments were performed. *P < 0.05 vs. NBT and LGG

Real-time polymerase chain reaction (RT-PCR) showed greatly reduced RIZ1 expression in HGGs, whereas R1Z1 was readily detectable in NBT and low-grade gliomas (LGGs) (Fig. 1a). Western blotting analysis demonstrated undetectable levels of R1Z1 expression in HGGs and a downregulation of RIZ1 protein expression in LGGs compared with NBT (Fig. 2a, b). Therefore, the level of RIZ1 expression was negatively correlated with the grade of gliomas. These results were replicated in human GBM (WHO IV) cell lines U87 and U251 (Fig. 1a and Fig. 2c,d).

### RIZ1 immunohistochemical expression in gliomas

**Fig. 3:**
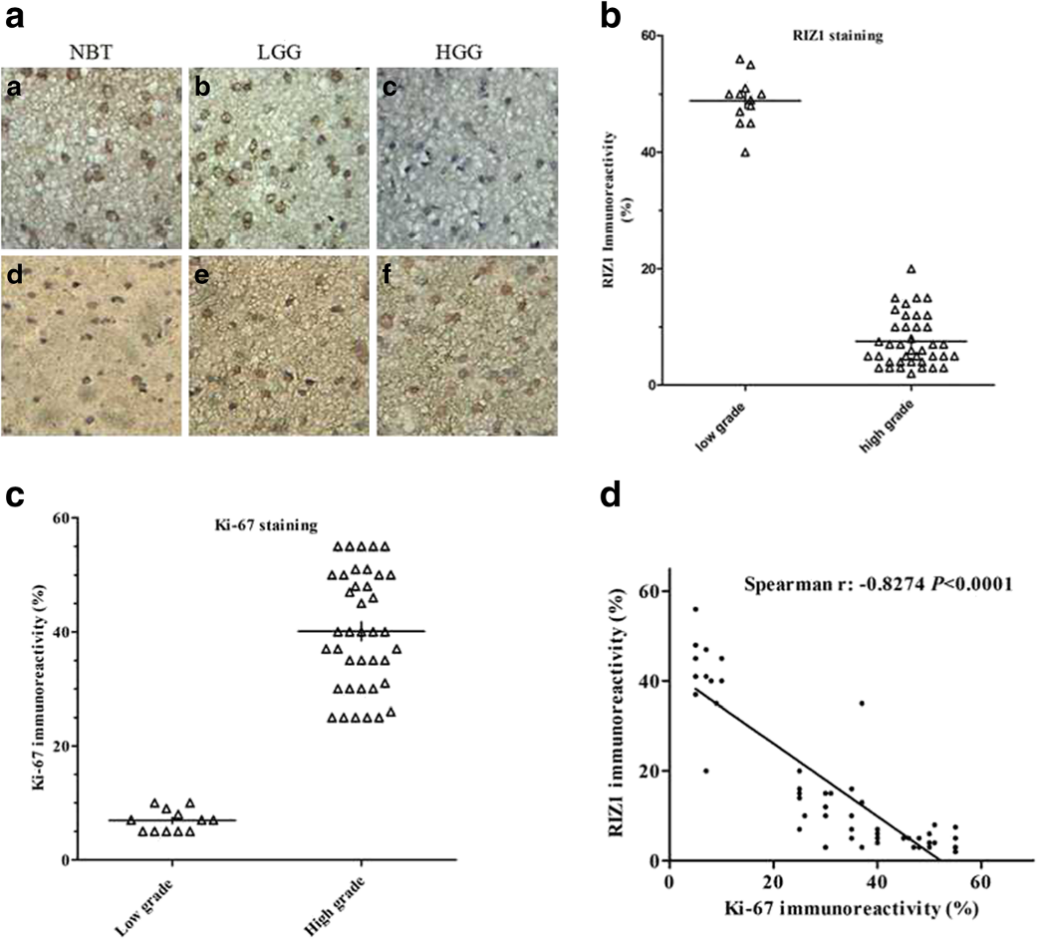
Immunohistochemical expression of RIZ1 (**a**: A–C) and Ki-67 (**a**: D–F) in NBT and gliomas. Quantitative analysis of RIZ1 (**b**) and Ki-67 (**c**) immunohistochemical expression in gliomas. The correlation between amount of RIZ1 and Ki-67 labeling (**d**)

Immunohistochemistry showed strong RIZ1 staining in the cytoplasm of NBT and LGGs, whereas a progressive loss of cytoplasmic RIZ1 staining was detected in HGGs (Fig. 3a). Statistical analysis confirmed reduced RIZ1 labeling in HGGs compared with LGGs (7.55 ± 4.39 % vs. 48.83 ± 4.34 %, *p* < 0.0001; Fig. 3b). Labeling of Ki-67, a proliferation marker detected in cell nuclei, was significantly higher in HGGs than in LGGs (40.10 ± 10.17 % vs. 6.92 ± 1.96 %, *p* < 0.0001; Fig. 3c). RIZ1 and Ki-67 immunoreactivity were negatively correlated (Spearman’s *r* = -0.8274, *p* < 0.0001; Fig. 3d).

### Correlation between RIZ1 immunoreactivity and clinicopathological features

To determine whether RIZ1 expression is associated with clinicopathological parameters, we performed correlations between RIZ1 expression and patient age, tumor histological grading, and other clinical features (Table 1). We found strong negative correlations between RIZ1 labeling and tumor grade (*p* < 0.0001) and patient age (*p* = 0.027).

### Relationship between RIZ1 immunoreactivity and patient prognosis

Kaplan-Meier survival analysis revealed a significant positive correlation between RIZ1 immunoreactivity and PFS (*p* < 0.0001; Fig. 1b). The probability of OS was also higher for glioma patients who exhibited higher RIZ1 expression (*p* < 0.0001) (Fig. 1c). Univariate survival analysis revealed significant relationships between RIZ1 expression, Ki-67 expression, and patient prognosis, whereas no significant associations were found between prognosis and patient age, gender, tumor size, or extent of resection (Table 2). Multivariate analysis using a Cox proportional hazards model with all the variables included in the univariate analysis revealed that high RIZ1 expression was an independent prognostic factor for patients with glioma (Table 3). Together, these results suggest that RIZ1 expression is a key predictor of survival in patients with gliomas.

**Table 2:** Kaplan-Meier univariate survival analysis of the relationship between clinicopathologic features and patient prognosis

Variables	Median Survival (months, 95 % CI)		*P*-values (log-rank)	Multi-p
RIZ1 (<Median vs. ≥Median)	12.000(9.324-14.676)	36.000(32.378-39.622)	<0.0001	0.000
Ki-67 (<Median vs. ≥Median)	36.000(32.814-39.186)	12.500(7.314-17.686)	0.001	0.202
Age (<50 vs. ≥50 years)	29.100(1.272-56.928)	16.000(6.600-25.400)	0.133	0.679
Gender (Male vs. Female)	14.000(9.149-18.851)	33.800(22.536-45.064)	0.469	NA
Diameter (≥6 cm vs. < 6 cm)	13.300(0.000-29.135)	28.000(7.468-48.532)	0.710	NA
Extent of resection (Gross vs. Sub)	23.8(7.158-40.442)	12.000(8.318-15.682)	0.706	NA

**Table 3:** Multivariate analysis using the Cox proportional hazards revealed that expression level of RIZ1 is an independent prognostic factors for patients with gliomas

variable		B	SE	Wald	df	Sig.	Exp(B)	95.0 % CI for Exp(B)	
								Lower	Upper
	RIZ1	-1.808	0.426	17.997	1	0.000	0.164	0.071	0.378

### Induced expression of RIZ1 in glioma cells inhibits cell proliferation and induces G2-M arrest

**Fig. 4:**
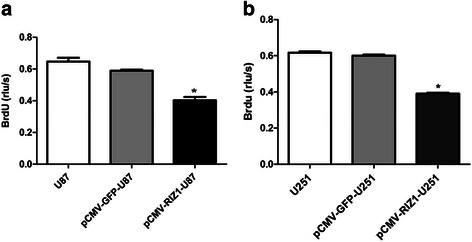
Induced expression of RIZ1 in U87 (**a**) and U251 (**b**) cells inhibited cell proliferation as measured by BrdU incorporation. The rlu/s in the Y axis represents the relative light units/second. Three independent experiments were performed. *P < 0.05 as compared with control groups

**Fig. 5:**
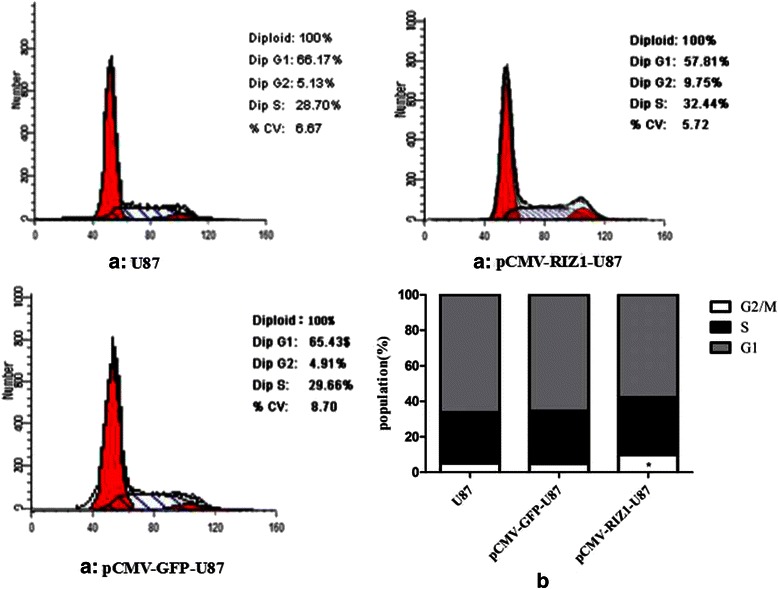
Effect of pCMV-RIZ1 on cell cycle in U87 cells. The data are the mean ± SD of one representative experiment. Similar results were obtained in three independent experiments. **b** statistical analysis of (**a**). *P < 0.05 vs control group

**Fig. 6:**
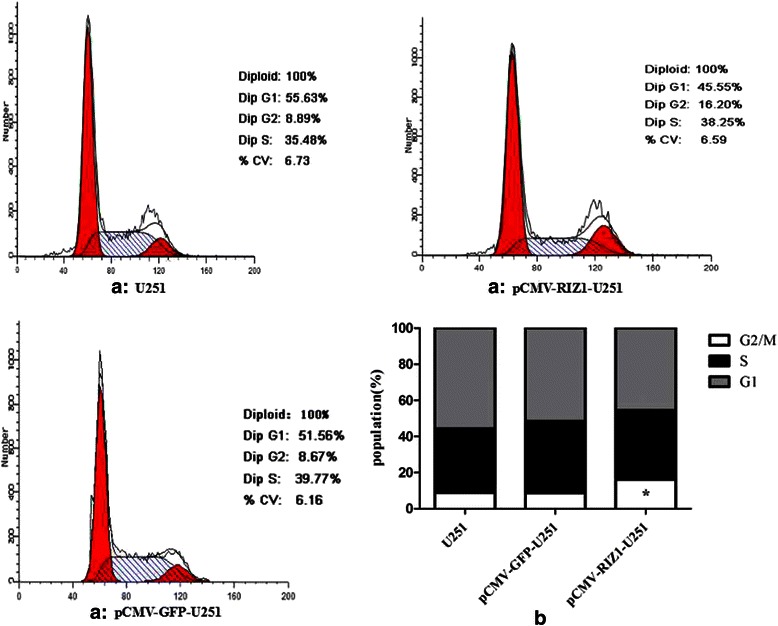
Effect of pCMV-RIZ1 on cell cycle in U251 cells. The data are the mean ± SD of one representative experiment. Similar results were obtained in three independent experiments. **b** statistical analysis of (**a**). *P < 0.05 vs control group

Next, we investigated the ability of RIZ1 to suppress growth of the glioblastoma cell lines U87 and U251. Transfection of pCMV-RIZ1 into U87 and U251 cells reduced both BrdU incorporation. However, no reduction of BrdU incorporation were observed after transfection with pCMV-green fluorescent protein (GFP) (Fig. 4). Further cell cycle analysis revealed that a majority of cells were arrested at G2-M 72 h after pCMV-RIZ1 infection (Figs. 5 and 6). By contrast, pCMV-GFP-infected cells showed no differences from parental U87 and U251 cells. Therefore, these results suggest that RIZ1 inhibits cell proliferation by inducing cell cycle arrest at the G2-M phase.

### Overexpression of RIZ1 in U87 and U251 cells induces apoptosis

**Fig. 7:**
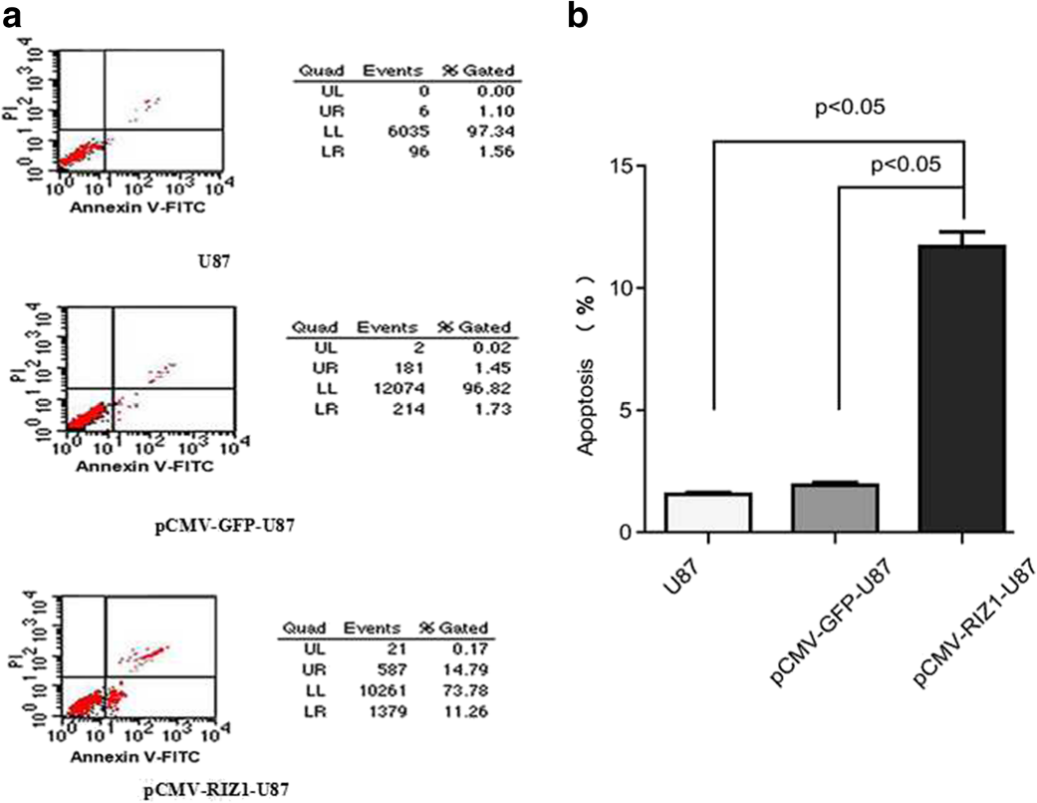
Effect of pCMV-RIZ1 on cell apoptosis in U87 cells. The data are the mean ± SD of one representative experiment. Similar results were obtained in three independent experiments. **b** statistical analysis of (**a**). *P < 0.05 vs control group

**Fig. 8:**
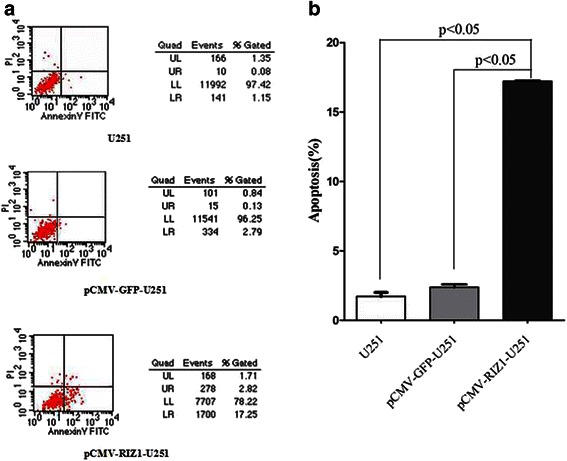
Effect of pCMV-RIZ1 on cell apoptosis in U251 cells. The data are the mean ± SD of one representative experiment. Similar results were obtained in three independent experiments. **b** statistical analysis of (**a**). *P < 0.05 vs control group

**Fig. 9:**
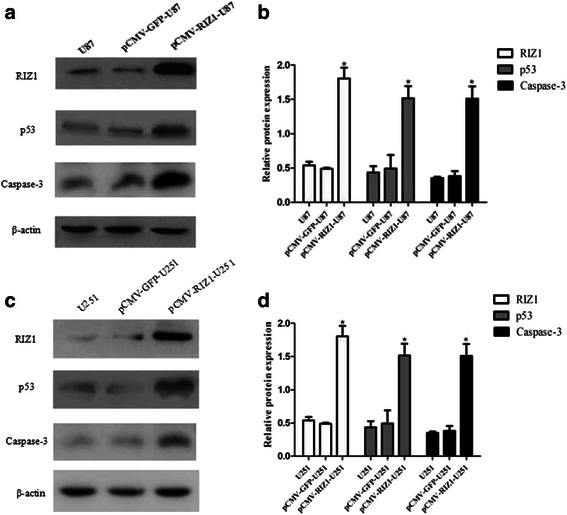
Western blotting analysis of pCMV-RIZ1 transfection for 72 h on p53, and caspase-3 expression in U87 (**a**) and U251 (**c**) cells. **b**, **d** Quantification of (**a, c**). *P < 0.05 vs control group

**Fig. 10:**
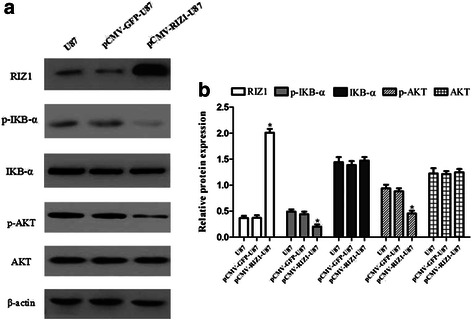
Western blotting analysis of pCMV-RIZ1 transfection for 72 h on p-IKBα, IKBα, p-AKT and AKT expression in U87 cells. **b** Quantification of (**a**). *P < 0.05 vs control group

**Fig. 11:**
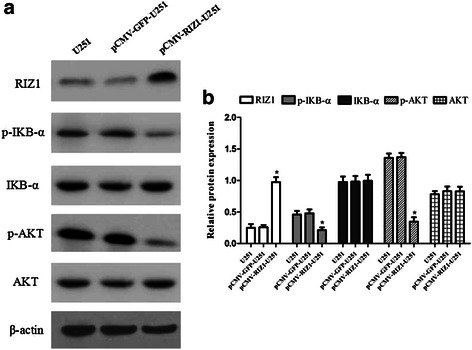
Western blotting analysis of pCMV-RIZ1 transfection for 72 h on p-IKBα, IKBα, p-AKT and AKT expression in U251 cells. **b** Quantification of (**a**). *P < 0.05 vs control group

Finally, we began to explore the effect of RIZ1 overexpression on apoptosis in U87 and U251 cell lines. The number of apoptotic U87 and U251 cells were significantly increased by transfection with pCMV-RIZ1 (Figs. 7 and 8). To further investigate the molecular mechanism by which RIZ1 inhibits glioma cell growth or induces apoptosis, we investigated whether RIZ1 affects the expression of p53. When transfected with pCMV-RIZ1, U87 and U251 cells exhibited elevated p53 and caspase-3 expression levels (Fig. 9). Furthermore, protein levels of p-IKBα and p-AKT were significantly lower in transfectants with higher RIZ1 expression as compared with control groups, whereas expression level of total IKBα and AKT were unchanged (Figs. 10 and 11). Therefore, RIZ1 may stimulate p53-mediated apoptosis in U87 cells and inhibit p-IKBα and p-AKT signaling pathways in glioma pathogenesis, although the specific pathways involved need further investigation.

## Discussion

RIZ1 is tightly linked to the progression of various types of cancers, as mutation, deletion, and altered DNA methylation of RIZ1 has been found in several human cancers [11–13]. Similar to p53, RIZ1 is a potential tumor suppressor that could serve as a novel target of cancer therapy. Previous study using PCR/SSCP found RIZ1 was not the target-gene mapped at 1p involved by mutation in oligodendroglioma development [14]. However, the expression level of RIZ1 in HGGs and its role in glioma pathogenesis remain unclear.

The present study demonstrates that RIZ1 expression was significantly inhibited in glioma samples (both HGGs and LGGs) compared with NBT. Considering glioma tissue, HGGs showed a greater reduction in RIZ1 levels than LGGs. This pattern of RIZ1 expression suggests the potential function of RIZ1 as a tumor suppression gene. Furthermore, HGGs showed a greater loss of cytoplasmic RIZ1 staining than LGGs, which agrees with previous studies using other human tumor samples [7–9]. We also found that RIZ1 expression was negatively associated with expression of Ki-67, which is known to be involved in cell proliferation. Thus, our results suggest that RIZ1 is a potent suppressor of gliomas. Additionally, Kaplan-Meier survival and multivariate analyses indicated a role for RIZ1 as a prognostic factor for glioma. Collectively, therefore, RIZ1 may be an important factor in the inhibition of glioma progression, and its expression could be used as a predictor of patient prognosis.

The mechanism of RIZ1 in cancer suppression had been widely studied. The most popular hypothesis is that RIZ1 causes G2-M arrest and apoptosis via cooperation with p53 [7–9]. Nest, we chosen U87 and U251 cell lines for the functional studies. Because U87 and U251 could represent the behavior of HGGs and they were the most available and deeply studied cell lines in our lab. And we have done lots of serial explorations in them with different genes and got excellent results [15–17]. Consistent with above hypothesis, we found that induced expression of RIZ1 in U87 and U251 cells inhibited cell proliferation, increased the number of apoptotic cells, and arrested cells at the G2-M phase. Furthermore, previous studies show that simultaneous RIZ1 and p53 deficiencies promote tumor formation in both mice and humans [18, 19], and DNA methylation of RIZ1 gene may be associated with nuclear accumulation of p53 [20]. Additionally, many sporadic human cancers are associated with both p53 mutations and RIZ1 silencing [7, 18]. One recent paper reported a tight relationship between RIZ1 and p53 due to a positive feedback circuit [21]. RIZ1 as a tumor regulatory protein is very likely to be involved in the regulation of inflammatory responses. Noman AS et al [21] reported that LPS significantly augmented the expression of RIZ1 and the augmentation was mediated by the activation of nuclear factor (NF)-κB and Akt. However, in our study, we found that RIZ1 stimulated p53-mediated apoptosis in U87 and U251 cells and inhibited p-IKBα and p-AKT signaling pathways. The possible reason is that the role of RIZ1 in inflammation response and cancer progression is quite different, and even the opposite. Future studies are required to clarify the interaction between RIZ1 and p53, and other signaling pathways in glioma pathogenesis.

## Conclusions

Our study provides direct evidence that RIZ1 is tightly associated with glioma malignancy. We believed that RIZ1 inhibits glioma progression and thus could be a potential therapeutic target for patients with gliomas.

## Abbreviations

HGG: high grade glioma
LGG: low grade gliomas
NBT: normal brain tissue
OS: overall survival
PFS: progress free survival
