# Erratum to: RIZ1: a potential tumor suppressor in glioma

**DOI:** 10.1186/s12885-016-2067-x

**Published:** 2016-01-18

**Authors:** Chenran Zhang, Qiubei Zhu, Hua He, Lei Jiang, Qiang Qiang, Liuhua Hu, Guohan Hu, Ying Jiang, Xuehua Ding, Yicheng Lu

**Affiliations:** Department of Neurosurgery, Changzheng Hospital, Second Military Medical University, Shanghai, 200003 China; Department of Otolaryngology, Changzheng Hospital, Second Military Medical University, Shanghai, 200003 China; Department of Neurology, Huadong Hospital, Fudan University, Shanghai, 200040 China; Department of Cardiology, Ren Ji Hospital, School of Medicine, Shanghai Jiao Tong University, Shanghai, 200127 China

## Erratum

Unfortunately, the original version of this article [1] contained an error in Fig. 9. Fig. 9b was used for both 9b and 9d and 9d was not included. The correct version of Fig. 9 can be found below.

**Fig. 9:**
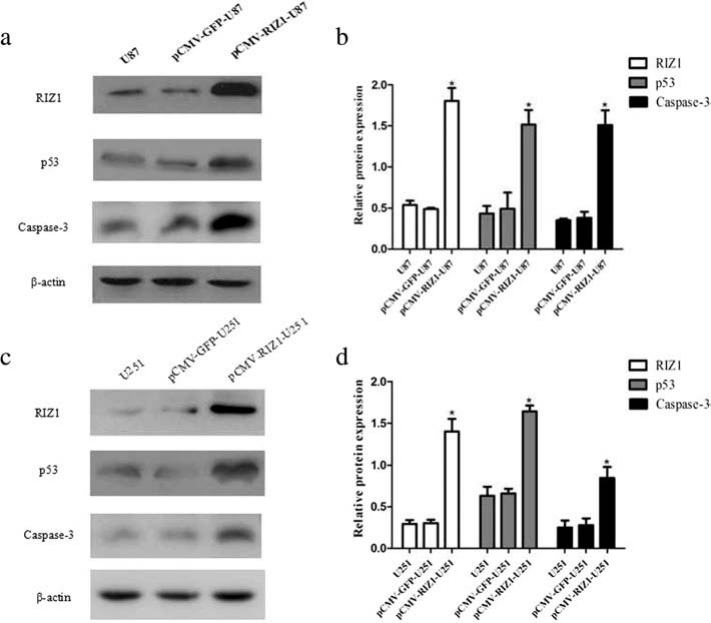
Western blotting analysis of pCMV-RIZ1 transfection for 72 h on p53, and caspase-3 expression in U87 (**a**) and U251 (**c**) cells. **b**, **d** Quantification of (**a**, **c**). *P < 0.05 vs control group
